# Brain-Dependent Processes Fuel Pain-Induced Hemorrhage After Spinal Cord Injury

**DOI:** 10.3389/fnsys.2019.00044

**Published:** 2019-09-10

**Authors:** Joshua A. Reynolds, Melissa K. Henwood, Joel D. Turtle, Rachel E. Baine, David T. Johnston, James W. Grau

**Affiliations:** Department of Psychological and Brain Sciences, Texas A&M University, College Station, TX, United States

**Keywords:** spinal cord injury, pain, hemorrhage, cytokines, caspase, nociception

## Abstract

Pain (nociceptive) input caudal to a spinal contusion injury can undermine long-term recovery and increase tissue loss (secondary injury). Prior work suggests that nociceptive stimulation has this effect because it fosters the breakdown of the blood-spinal cord barrier (BSCB) at the site of injury, allowing blood to infiltrate the tissue. The present study examined whether these effects impact tissue rostral and caudal to the site of injury. In addition, the study evaluated whether cutting communication with the brain, by means of a rostral transection, affects the development of hemorrhage. Eighteen hours after rats received a lower thoracic (T11–12) contusion injury, half underwent a spinal transection at T2. Noxious electrical stimulation (shock) was applied 6 h later. Cellular assays showed that, in non-transected rats, nociceptive stimulation increased hemoglobin content, activated pro-inflammatory cytokines and engaged signals related to cell death at the site of injury. These effects were not observed in transected animals. In the next experiment, the spinal transection was performed at the time of contusion injury. Nociceptive stimulation was applied 24 h later and tissue was sectioned for microscopy. In non-transected rats, nociceptive stimulation increased the area of hemorrhage and this effect was blocked by spinal transection. These findings imply that the adverse effect of noxious stimulation depends upon spared ascending fibers and the activation of rostral (brain) systems. If true, stimulation should induce less hemorrhage after a severe contusion injury that blocks transmission to the brain. To test this, rats were given a mild, moderate, or severe, injury and electrical stimulation was applied 24 h later. Histological analyses of longitudinal sections showed that nociceptive stimulation triggered less hemorrhage after a severe contusion injury. The results suggest that brain-dependent processes drive pain-induced hemorrhage after spinal cord injury (SCI).

## Introduction

Spinal cord injuries are frequently accompanied by additional tissue damage (polytrauma) that can provide a source of pain (nociceptive) input after injury (Saboe et al., 1991; Chu et al., 2009). This is clinically important because research has shown that nociceptive stimulation caudal to spinal cord injury (SCI) can sensitize pain circuits, impair adaptive plasticity, increase tissue loss (secondary injury) at the site of injury, and undermine long-term recovery (Grau et al., 2017).

Our laboratory has studied the adverse effect of nociceptive input on recovery using animals that have undergone a lower thoracic (T11–12) contusion injury (Grau et al., 2004). Pain fibers are engaged using intermittent electrical stimulation (shock) applied at a intensity and duration known to impair adaptive plasticity in spinally transected animals (Grau et al., 1998; Crown et al., 2002). Using this paradigm, we have shown that just 6 min of uncontrollable shock applied to the tail or hind leg a day after injury impairs long-term recovery and increases tissue loss. Noxious stimulation also delays the recovery of bladder function and fosters the development of spasticity and chronic pain (Grau et al., 2004). Application of the irritant capsaicin, which engages unmyelinated nociceptive (C) fibers that express the transient receptor potential cation channel subfamily V member 1 (TRPV1) receptor, also impairs long-term recovery (Turtle et al., 2018). Research has shown that noxious stimulation has an especially robust effect if given within 4 days of injury (Grau et al., 2004). The acute effect of engaging nociceptive fibers has been linked to the expression of pro-inflammatory cytokines [e.g., tumor necrosis factor (TNF), interleukin-1β (IL-1β), and IL-18] and the activation of cellular signals (e.g., caspase 1, 3, 8) related to cell death (Garraway et al., 2011, 2014; Lossi et al., 2015; Grau and Huang, 2018; Turtle et al., 2018).

New data suggest that nociceptive input (from electrical stimulation or the application of capsaicin) caudal to a contusion injury increases tissue loss (secondary injury) because it leads to a breakdown of the blood-spinal cord barrier (BSCB; Turtle et al., 2017, 2019). The development of this effect is associated with capillary fragmentation and the *de novo* formation of the sulfonylurea receptor 1-transient receptor potential melastatin 4 (SUR1-TRPM4) cation channel, two pathogenic features of progressive hemorrhagic necrosis (Simard et al., 2013). Because blood borne cells and proteins are neurotoxic (Mautes et al., 2000), the infiltration of blood will expand the area of injury and undermine long-term recovery. To date, our exploration of these effects has been limited to a 1-cm region of the spinal cord that encompasses the area of injury. The present study uses a combination of cellular assays and histology to evaluate whether noxious stimulation has an effect that extends beyond this area to affect hemorrhage and cytokine expression rostral and caudal to the site of injury.

We also explored whether communication with the brain influences the development of hemorrhage in response to noxious electrical stimulation. To explore this issue, animals underwent a second surgery that transected the spinal cord at T2, rostral to the T11/12 contusion injury. Prior work has shown that electrical stimulation at an intensity that engages C-fibers, or chemically engaging pain fibers with capsaicin, sensitizes nociceptive processes within the lumbosacral spinal cord and impairs adaptive learning in spinally transected rats (Grau et al., 1998, 2012, 2014; Crown et al., 2002; Ferguson et al., 2006, 2012; Baumbauer et al., 2008; Hook et al., 2008; Grau, 2014). Interestingly, noxious stimulation does not induce an alteration in spinal processing in uninjured (intact) animals (Washburn et al., 2007). Likewise, electrical stimulation of the sciatic nerve can induce long-term potentiation (LTP) in spinally transected, but not intact, animals (Sandkühler, 2000, 2009). These observations suggest that descending fibers can quell over-excitation, which would be expected to mitigate the adverse effect noxious input has on tissue survival after a contusion injury. From this perspective, cutting communication with the brain should amplify the effect of nociceptive input, fueling cytokine expression and hemorrhage at the site (T11/12) of injury. Contrary to our expectations, our data demonstrated exactly the opposite—a rostral T2 transection blocked the effect noxious electrical stimulation. Recognizing the novelty of this finding, we replicated the finding using histological procedures to evaluate the extent of hemorrhage. The results imply that surviving ascending fibers engage brain-dependent processes that fuel tissue loss at the site of injury. If this is true, increasing the severity of the contusion injury should weaken pain signaling to the brain and thereby mitigate the adverse effect of noxious stimulation applied caudal to injury. Our last experiment provides evidence for this type of interaction.

## Materials and Methods

### Animals

Male Sprague–Dawley rats were purchased from Envigo (Houston, TX, USA) and were acclimated to housing for at least 7 days prior to surgery. Animals (308 and 373 g) were dual housed with water and food *ad libitum* and maintained on a 12-h light-dark cycle. Behavioral testing and surgeries were performed during the light portion of the cycle. All experiments and procedures followed the NIH standards for the care and use of laboratory animals (NIH publication No. 80-23) and were approved by the Institutional Animal Care and Use Committee (IACUC) at Texas A&M University. A power analysis was used to determine the minimum number of animals needed to achieve statistical significance, and every effort was made to minimize pain and suffering.

### Contusion Injury

Rats were anesthetized with 5% isoflurane using an induction chamber. An area approximately 6 cm wide was shaved from the base of the skull to the tail. After shaving, the surgery area was sterilized with iodine and 70% ethanol. Anesthesia was maintained with 2–4% isoflurane administered through a nose cone. A 7 cm longitudinal incision was made through the skin centered around the twelfth thoracic vertebra (T12). Next, an incision was made through the musculature on either side of the spinal column cut to the depth of just above the rib cage. Soft tissue was then removed from the dorsal surface of the spinal column with rongeurs and a laminectomy was performed on the T12 vertebra and the caudal half of the T11 vertebra prior. Prior to injury, the surgery area was flushed with sterile saline to remove debris. The MASCIS device was used to perform the contusion injury (Gruner, 1992). Clamps on the MASCIS device were inserted into the previously made incisions, and the subjects were suspended by their spine. The spine was aligned and pulled taut prior centering the impactor over the exposed cord. In the first two experiments, a moderate contusion injury was performed by dropping the 10 g impactor (2.5 mm head) from a height of 12.5 mm directly onto the exposed cord. In the last experiment, the impactor height was set at 0 (no impact), 6.25, 12.5, or 25 mm. Following contusion, the surgery site was closed with Michel clips. Subjects were administered 100,000 units/kg of penicillin and 3 mL of saline after surgery through intraperitoneal injection to prevent infection and account for blood loss that occurred during the procedure.

### Spinal Transection

Half of the animals in first two experiments also had the spinal cord transected (Trans) at the second thoracic vertebra (T2). In the first, the spinal cord was transected 18 h after the contusion injury while in the second it was transected immediately after, when animals were still anesthetized. For the remote transection (at 18 h after the contusion injury) anesthesia was induced as described above, with 5% isoflurane in an induction chamber. Thereafter, anesthesia was maintained with 2–4% isoflurane applied through a nose cone. To transect the spinal cord, a 3 cm incision was made starting 2 cm rostral to the T2 vertebra and extending 1 cm caudal. A V-cut was made through the musculature originating just rostral to the T2 vertebra and extending caudally to isolate the spinous process of the T2 vertebra. Spreaders were used to open the surgery site and the spinous process of the T2 vertebra was followed down to the body of the vertebra. Soft tissue was cleared between the T1 and T2 vertebrae, exposing the cord, and a spinal transection was performed immediately rostral to the T2 vertebra. Transections were performed with the Thermal Cautery Unit, manufactured by Geiger. The surgical incision was closed with Michel clips and animals were administered 3 ml of saline following surgery. The remaining rats underwent a sham surgery. For these animals, the procedure was identical except the spinal cord was not transected. Transections were verified by visual inspection of the site during surgery and a post-mortem examination of the spinal cord.

### Nociceptive Stimulation

Animals were loosely restrained in opaque Plexiglass tubes housed in an acoustic isolation chamber. Electrical stimulation was applied through tail electrodes formed from a modified fuse clip, as described in Grau et al. (1998). Briefly, the electrodes were coated with electrode gel (Harvard Apparatus, Holliston, MA, USA) and attached 2 cm from the tip of the tail with Orthaletic tape. The electrodes were connected to a BRS/LVE shock generator (Model SG-903), and constant current 1.5-mA, AC (60 Hz) electrical stimuli (100 ms in duration) was applied on a variable intermittent schedule (0.2–3.8 s; rectangular distribution) for 6 min. Prior work has shown that this level of stimulation expands the region of secondary injury, promotes hemorrhage, and impairs long-term recovery (Grau et al., 2004; Turtle et al., 2017, 2019). Unshocked controls were treated the same except shock was withheld.

### Assessment of Recovery

In the last experiment, locomotor function was assessed using the scoring system developed (BBB) by Basso et al. (1995) while animals explored an open field. Performance was assessed prior to (−1 h) shock treatment, or equivalent period of restraint (Unshock). Motor behavior was re-assessed immediately after (0 h) and again 1, 2, and 3 h later. Care was taken to assure that individuals performing behavioral scoring had high inter-observer reliability (>95%) and were unaware of treatment condition.

### Tissue Collection

Animals were euthanized with pentobarbital (100 mg/kg) 3 h after animals had received shock or an equivalent period of restraint (Unshock). Tissue in the first experiment was collected and flash-frozen in liquid nitrogen. Tissue collected included a 1 cm region that encompassed the site of injury. In addition, we obtained 1 cm of tissue rostral and caudal to the site of injury.

In the next two experiments, animals were euthanized and a 3 cm segment of the spinal cord, centered at the site of injury, was collected. After the heartbeat had terminated, subjects were perfused transcardially with ~300 mL of ice-cold phosphate-buffered saline (PBS; pH 7.3) followed by ~400 mL of 4% paraformaldehyde (PFA). Spinal cord tissue at the site of injury was collected and incubated in 4% PFA for 24 h at 4°C. The tissue was rinsed with PBS before cryoprotection in a solution of 30% sucrose in PBS for at least 72 h.

### Cellular Assays

Tissue was prepared as described in Garraway et al. (2014). Briefly, the cord was processed for the extraction of both total RNA (RNeasy Mini Kit; Qiagen, Valencia, CA, USA) and total protein. After RNA extraction, total protein was extracted from the organic layer using the QIAzol lysis reagent protocol for isolation of genomic DNA and/or proteins from fatty tissue (Qiagen, Valencia, CA, USA). A Bradford assay (BioRad, Hercules, CA, USA) was used to determine the concentration of protein extracts. Protein samples were diluted in 4× Laemmli buffer to a final concentration of 3 mg/mL.

Spectral analyses for free hemoglobin were conducted using protein extracts from the lesioned tissue. Spectrophotometric absorbance was measured from 200 to 800 nm from 1.0 μL of protein extract (NanoDrop, Thermo Scientific). Absorbance at 420 nm was used as a measure of hemoglobin content (Turtle et al., 2019).

Western blot analysis was then used to quantify TNF, caspase-1, and 3, IL-1β, and IL-18 as described in Turtle et al. (2018). Briefly, samples were transferred onto PVDF membranes (Millipore, Bedford, MA, USA), the blots were blocked for 1 h in 5% blotting-grade milk (BioRad, Hercules, CA, USA) in Tris-buffered saline Tween-20 (TBST). After blocking, the blots were incubated overnight at 4°C in one of the following primary antibodies generated in rabbit: TNFα (1:500; #ARC3012—Invitrogen, Camarillo, CA, USA; AB_305641), caspase-1 (1:1,000; #ab1872—Abcam, Cambridge, MA, USA; AB_302644), caspase-3 (1:1,500; #NB600-1235—Novus Biological, Littleton, CO, USA; AB_2069897), IL-1β (1:200; #sc-7884—Santa Cruz Biotechnology, Santa Cruz, CA, USA; AB_2124476), IL-18 (1:200; #sc-7954—Santa Cruz Biotechnology, Santa Cruz, CA, USA; AB_1564060), or lamin B (1:1,000; #ab16048—Abcam, Cambridge, MA, USA; AB_443298). The next day, blots were washed in TBST (3 × 10 min) at room temperature then incubated in HRP-conjugated goat anti-rabbit secondary antibodies (1:5,000; #31460; Pierce, Rockford, IL, USA) for 1 h at room temperature. After another 3 × 10 min series of washes, the blots were developed with electrochemiluminescence (Pierce, Rockford, IL, USA) and imaged with Fluorchem HD2 (ProteinSimple, Santa Clara, CA, USA). Ratios of the integrated densitometry of each protein of interest to the loading control (lamin B) were calculated and normalized to a control group (run on the same blot) that did not receive nociceptive stimulation.

### Microscopy

To quantify hemorrhage in the second experiment, collected spinal cords were sectioned into evenly spaced 20 μm-thick coronal sections in the 3 cm of cord surrounding the injury site. Sections were mounted on Fisherbrand Superfrost Plus (Fisher Scientific) microscope slides.

The tissue was stained with hematoxylin and eosin (H&E) as previously described (Turtle et al., 2019). Sections were washed with distilled water to remove residual tissue, then incubated in hematoxylin for 4 min. Slides were then dipped in acid alcohol (1% hydrochloric acid in 70% ethanol) twice. Next, slides were incubated in Scott’s tap water (Sigma-Aldrich, Cat# S5134) substitute for 1 min. Finally, slides were incubated in 70% eosin for 1 min, and dehydrated with ethanol and xylene before mounting with Permount. Sections were imaged using light microscopy at 4× magnification and were analyzed by blinded observers using ImageJ software. Hemorrhaged areas appeared substantially redder than surrounding tissue, which was confirmed by the presence of red blood cells at a higher magnification. To determine the total area of hemorrhage, the affected region was traced by a blinded observer. The amount of hemorrhage was quantified as a percentage of the total section area.

For the last experiment, the impact of injury severity on hemorrhage was assessed by taking 3 cm longitudinal sections of spinal cord centered on the site of injury. Sections (20 μm) were collected along the dorsal-ventral axis and mounted on microscope slides. Sections were prepared and stained as described above. For each animal, the section set was divided evenly into three clusters (dorsal, medial, and ventral) and the median section from each was selected for analysis. The extent of hemorrhage was analyzed at 2× magnification using ImageJ software to assess the area of hemorrhage. The amount of hemorrhage was assessed as a percentage of the total section area.

### Experimental Designs

To evaluate the rostral/caudal spread of hemorrhage and cytokine expression, rats received lower thoracic (T11–12) contusion injury. To determine whether brain systems influence how noxious input affects these processes, half the animals under went a second surgery to transect (Trans) the spinal cord at T2 18 h after the contusion injury. The remaining rats received a laminectomy at T2, but the spinal cord was not transected (Sham). Six hours later, animals were placed in the restraining tubes and prepared for tail shock. Half of the rats in each surgery condition (*n* = 6) then received 6 min of intermittent shock (Shock). The remaining rats served as the unshocked controls (Unshock). Three hours later, a 1 cm segment of tissue that encompassed the injury, as well as 1 cm segments rostral and caudal, were collected. Hemorrhage was assessed using spectrophotometry and Western blotting for alpha hemoglobin. Western blotting was also used to assess the expression of key cytokines (TNF, IL-1β, IL-18) and signals related to cell death (caspase 1, 3; Lossi et al., 2015). The complete experiment involved a 2 (Sham vs. Trans) × 2 (Shock vs. Unshock) factorial and used 24 rats (*n* = 6).

Next, histology was used to evaluate the rostral/caudal spread of hemorrhage and the effect of cutting communication with the brain. Immediately after the spinal cord was contused at T11–12, half of the rats had the spinal cord transected at T2 (Trans). The remaining animals underwent a laminectomy at T2, but the spinal cord was not transected (Sham). Twenty-four hours later, the animals were placed in restraining tubes and given shock, or nothing (Unshock), as described above (*n* = 4). Three hours later, a 3-cm length of the spinal cord that encompassed the area of injury was collected and prepared for histology. Coronal tissue sections were collected at the injury center and ±0.17, 0.35, 0.52, and 1.02 cm and stained with H&E. The area of hemorrhage was quantified using ImageJ software by researchers who were blinded to treatment condition. The experiment involved 2 (Sham vs. Trans) × 2 (Shock vs. Unshock) factorial and used 16 rats (*n* = 4).

Finally, we evaluated whether the effect of pain input on hemorrhage varies with injury severity. Rats received a contusion injury with the impactor height set at 0, 6.25, 12.5, or 25 mm for a sham, mild, moderate, or severe injury, respectively. The next day, locomotor performance was assessed before animals were exposed to 6 min of intermittent electrical stimulation (Shock) applied to the tail or nothing (Unshock; *n* = 4). Locomotor behavior was assessed again immediately after stimulation and 1, 2, and 3 h later. Animals were then euthanized and 3-cm of the spinal cord, encompassing the area of injury, was collected and prepared for histology. Longitudinal sections were collected from the dorsal, medial, and ventral regions, stained with H&E, and the area of hemorrhage was quantified as described above. The experiment involved a 4 [injury severity (0, 6.25, 12.5 or 25 mm drop)] × 2 (Shock vs. Unshock) factorial and used 32 rats (*n* = 4).

### Statistics

All of the experiments employed full factorial designs and an equal number of animals per condition. Prior work has shown that the experimental treatments examined have a large effect size (*d* > 1.4). A power analysis confirmed that our sample sizes (4–6 per group) were sufficient to achieve statistical significance. Animals were randomly assigned to experimental treatments and the researchers conducting behavioral or cellular assays were blind to treatment condition. All data were analyzed using analysis of variance (ANOVA) or analysis of covariance (ANCOVA). For key terms, we also provide a measure of the proportion of variance accounted for [eta squared (*η*^2^)]. When necessary, *post hoc* comparisons of the group means were performed using Duncan’s New Multiple Range test. A Bonferroni *t*-test was used to evaluate group differences across region or time. In all cases, a criterion of *p* < 0.05 was set as the threshold for statistical significance.

## Results

### Spinal Transection Blocks the Effect of Nociceptive Stimulation on Hemorrhage

The first experiment evaluated whether nociceptive stimulation applied a day after animals received a lower thoracic contusion injury affects tissue rostral and caudal to the site of injury. The experiment also tested whether cutting communication with the brain, by means of an upper thoracic transection, would amplify the effect of stimulation on hemorrhage and pro-inflammatory cytokine expression.

**Figure 1:**
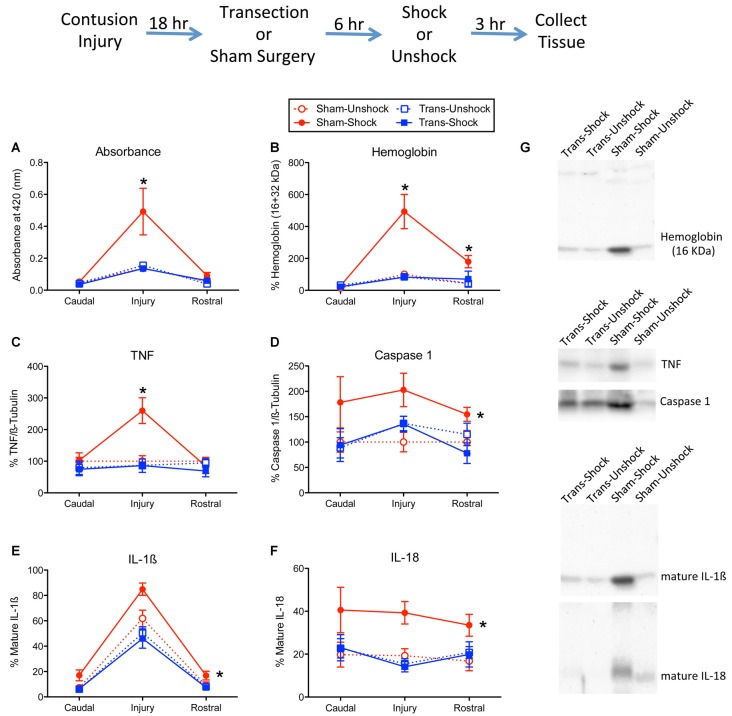
Impact of shock on indices of hemorrhage and signals linked to inflammation and cell death. The experimental design is illustrated at the top of the figure. Eighteen hours after rats received a lower thoracic (T11–12 vertebrae) contusion injury, animals received a T2 spinal transection (Trans) or sham surgery. Six hours later, animals were exposed to noxious electrical stimulation (Shock) to the tail, or nothing (Unshock) and 3 h later a 1 cm portion of the spinal cord that encompassed the injury was collected. Additional tissue, 1-cm rostral and caudal to injury, was also obtained and prepared for cellular assays. Protein samples from non-transected animals that received shock (Sham-Shock) exhibited greater **(A)** absorbance at 420 nm, the absorption peak for hemoglobin. Western blotting showed that animals in the Sham-Shock group had higher levels of **(B)** alpha hemoglobin, **(C)** tumor necrosis factor (TNF), **(D)** caspase 1, **(E)** interleukin-1β (IL-1β), and **(F)** IL-18 at the site of injury. Nociceptive stimulation had no effect in animals that had received a spinal transection (Trans-Shock). **(G)** Representative blots from the injury center are depicted to the right of the figures. Error bars indicate the standard error of the mean (SEM). An asterisk above an error bar indicates that the effect of nociceptive stimulation was significant for that region of the spinal cord (*p* < 0.05, *n* = 6). An asterisk placed to the right indicates that stimulation had a broader effect that did not vary across the regions assayed.

As previously reported (Grau et al., 2017; Turtle et al., 2019), in non-transected shocked rats (Sham-Shock) protein samples from the site of injury had a reddish tint. To quantify this effect, spectrophotometry was used to assess absorbance at 420 nm, the wavelength associated with hemoglobin. Non-transected rats that received intermittent shock a day after injury exhibited greater absorbance at the site of injury (Figure 1A). Surprisingly, this effect was blocked by spinal transection. An ANOVA confirmed that the main effects of surgery, pain treatment, and the surgery × shock treatment interaction, were statistically significant, all *F*s > 4.49, *p* < 0.0468 (all *η*^2^ > 0.136). In addition, the main effect of tissue region (caudal, injury, or rostral), and its interactions with surgery and shock treatment, were significant, all *F*s > 3.76, *p* < 0.0318. The three-way interaction indicates that the effect of shock treatment varied across tissue region (rostral, injury, or caudal) and surgery condition (sham vs. transected). *Post hoc* comparisons of the group means, collapsed across tissue region, showed that non-transected rats that received shock (Sham-Shock) differed from the other three groups (*p* < 0.05). No other group difference approached statistical significance (*p* > 0.05).

Western blotting for alpha hemoglobin confirmed that the extent of blood infiltration depended upon surgery and shock treatment (Figures 1B,G), both *F*s > 8.79, *p* < 0.0077 (both *η*^2^ > 0.185). Further, the effect of shock treatment interacted with surgery condition, *F*_(1,20)_ = 8.92, *p* = 0.0073 (*η*^2^ = 0.102). Comparisons of the group means showed that the non-transected group that received shock (Sham-Shock) differed from the other three (*p* < 0.05). No other group differences (collapsed across region) were significant (*p* > 0.05). Overall levels also varied across region, *F*_(2,40)_ = 22.75, *p* < 0.0001. The two-way and three-way interactions between region, surgery, and pain treatment were also statistically significant, all *F*s > 7.74, *p* < 0.0014. To further analyze the nature of this three-way interaction, we compared groups at each region using the Bonferroni *t*-test, which keeps the error rate at 0.05 for a family of contrasts. These analyses showed that the Sham-Shock group differed from the other three at, and rostral to, the site of injury (*p* < 0.05). No other comparisons were significant (*p* > 0.05).

Nociceptive stimulation has been shown to engage the proinflammatory cytokine TNF (Garraway et al., 2014). In the present study, we found that shock treatment increased TNF expression at the site of injury (Figure 1C). This yielded a significant interaction between region, surgery condition, and shock treatment, *F*_(2,40)_ = 4.12, *p* = 0.0237 (*η*^2^ = 0.102). The main effects of surgery and region, and their interaction, were also significant, all *F*s > 4.25, *p* < 0.0213. In addition, the effect of shock treatment varied with region, *F*_(2,40)_ = 6.03, *p* = 0.0051. No other term from the ANOVA reached statistical significance, all *F*s > 4.08, *p* > 0.05. *Post hoc* comparisons of the group means showed that the Sham-Shock group differed from the other three (*p* < 0.05). No other group comparison was significant (*p* > 0.05).

We previously showed that nociceptive stimulation increases the expression of caspase 1, which cleaves IL-1β and IL-18 to their active (mature) forms (Turtle et al., 2018). Here, we found that nociceptive stimulation augmented caspase 1 expression in the non-transected rats (Sham-Shock), but not transected rats (Trans-Shock; Figure 1D). An ANOVA verified that the effect of shock treatment depended upon surgery condition, *F*_(1,20)_ = 5.09, *p* < 0.0354 (*η*^2^ = 0.166). No other term was statistically significant, all *F*s < 2.91, *p* > 0.05. *Post hoc* comparisons of the group means confirmed that the Sham-Shock group differed from the other three (*p* < 0.05). No other group comparison was significant (*p* > 0.05).

Western blotting for the mature form of IL-1β revealed greater expression at the site of injury and that the magnitude of this effect was amplified by shock treatment (Figure 1E). Here too, the effect of shock treatment was blocked by spinal transection. An ANOVA confirmed that the effect surgery and shock treatment, and their interaction, were statistically significant, all *F*’s > 4.94, *p* < 0.0379 (all *η*^2^ > 0.0941). *Post hoc* comparisons of the group means showed that the non-transected group that received shock (Sham-Shock) differed from the other three (*p* < 0.05). No other group comparisons were significant (*p* > 0.05). The level of IL-1β expression also varied across region, *F*_(2,40)_ = 186.82, *p* < 0.0001, and the magnitude of this effect depended upon surgery condition (sham vs. transected), *F*_(2,40)_ = 6.98, *p* = 0.0025. The latter effect emerged because spinal transection attenuated the overall levels of IL-1β expression. No other term reached statistical significance, both *F*s < 1.50, *p* > 0.05.

The overall profile of expression for the mature form of IL-18 (Figure 1F) mirrored the pattern observed for caspase 1. An ANOVA showed that the effect of shock treatment depended upon surgery condition, *F*_(1,20)_ = 4.90, *p* < 0.0387 (*η*^2^ = 0.167). No other term was significant, all *F*s < 4.21, *p* > 0.05. *Post hoc* comparisons of the group means (collapsed across region) showed that the non-transected group that received shock (Sham-Shock) differed from the other three (*p* < 0.05). No other difference was statistically significant (*p* > 0.05).

We also assessed the active form of caspase 3 (17 kDa). Western blotting failed to detect any activity within the rostral or caudal regions. At the site of injury, non-transected rats that received shock exhibited higher expression [224.8% ± 26.6 (mean ± SE)] relative to the non-transected unshocked controls (100% ± 20.4). Expression was low in both shocked (68.7% ± 12.8) and unshocked (115% ± 18.2) transected rats. An ANOVA conducted on the tissue from the injury site confirmed that both surgery and its interaction with shock treatment were statistically significant, both *F*s > 12.18, *p* < 0.0023. The main effect of shock was not significant, *F*_(1,20)_ = 3.74, *p* > 0.05. *Post hoc* comparisons showed that the non-transected shocked (Sham-Shock) group differed from the other three (*p* < 0.05). No other group comparisons were significant (*p* > 0.05).

Because there was some evidence of hemorrhage and IL-1/IL-18/caspase 1 expression rostral to injury, we also assessed the levels of these proteins within the cervical region. In no case was there a significant effect of shock treatment or transection surgery, all *F*s < 2.10, *p* > 0.05.

### Histological Evidence that Spinal Transection Blocks Hemorrhage

The results reported above yielded an unexpected outcome, demonstrating that an upper (T2) thoracic transection blocks nociception-induced hemorrhage in contused rats. The implication is that pain input after a contusion injury only expands the area of hemorrhage if some communication with the brain is preserved. To gain converging evidence that a transection impacts hemorrhage, histological analyses were used to quantify hemorrhage extent. Because our cellular assays revealed some hemorrhage rostral to the site of injury, hemorrhage was assessed across 3 cm longitudinal sections of the spinal cord that encompassed the site of injury.

**Figure 2:**
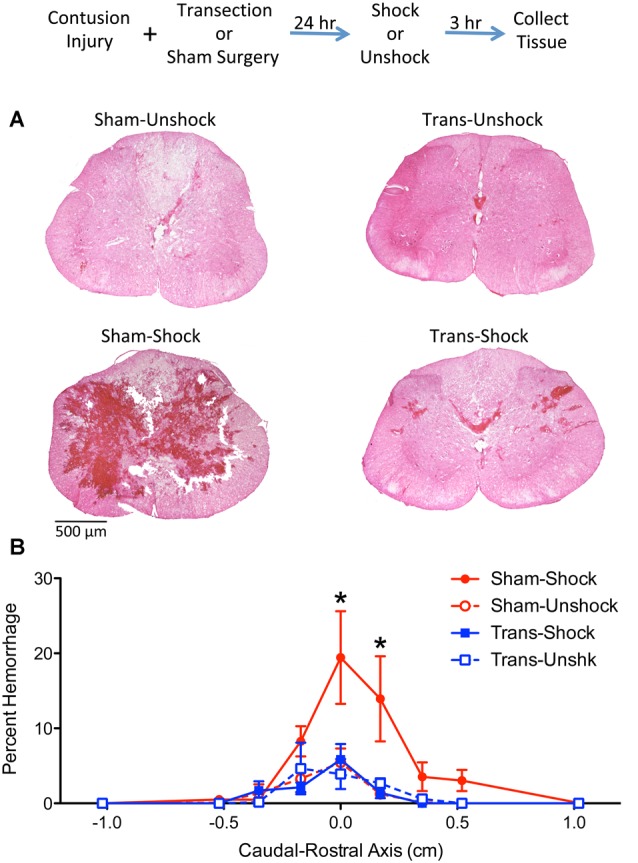
Noxious electrical stimulation increased the area of hemorrhage in non-transected animals. The experimental design is illustrated at the top of the figure. Rats received a lower thoracic (T11–12 vertebrae) contusion injury and a spinal transection (Trans) at T2 or sham surgery. A day later, half the animals were exposed to electrical stimulation (Shock) or nothing (Unshock). Tissue was collected 3 h later, sectioned, and stained with H&E. **(A)** Noxious stimulation increased the area of hemorrhage at the epicenter of injury in non-transected rats that received shock (Sham-Shock). Areas of hemorrhage appear darkly stained (bar = 500 μm). **(B)** Quantitation of hemorrhage extent over the caudal-rostral axis showed that the maximal effect was observed within 0.5 cm of the injury center and was positively skewed. Nociceptive stimulation did not amplify hemorrhage in spinally-transected rats (Trans-Shock). Error bars indicate the SEM. *Indicates statistical significance (*p* < 0.05, *n* = 4).

H&E stained sections from non-transected contused rats that received shock (Sham-Shock) showed extensive areas of hemorrhage at the epicenter of injury (Figure 2A). The area of hemorrhage was confined to less than 1-cm of tissue (Figure 2B) and skewed towards the rostral side of the injury. The effect of shock treatment was completely blocked by spinal transection (Trans-Shock). An ANOVA confirmed that the effect of shock treatment interacted with spinal transection, *F*_(1,12)_ = 4.811, *p* = 0.0487 (*η*^2^ = 0.187). The extent of hemorrhage varied across the caudal-rostral axis, *F*_(8,96)_ = 10.122, *p* < 0.0001, and this effect interacted with both spinal transection and shock treatment, all *F*s > 2.050, *p* < 0.0484. A three-way interaction emerged because animals in the Sham-Shock condition exhibited greater hemorrhage along the caudal-rostral axis. *Post hoc* comparisons of the group means, collapsed across the caudal-rostral axis, confirmed that the Sham-Shock group differed from the other three (*p* < 0.05). No other comparisons were significant (*p* > 0.05).

### Nociceptive Stimulation Induces Greater Hemorrhage After a Less Severe Injury

Our results provide further evidence that pain input caudal to a contusion injury can expand the area of hemorrhage (Grau et al., 2004, 2017; Turtle et al., 2019). Contrary to our original hypothesis, cutting communication with the brain eliminated this effect. This implies that the development of hemorrhage after injury is regulated by spared fibers. These observations suggest that the extent to which noxious stimulation induces hemorrhage will vary with injury severity, with a more robust effect being observed after less severe (mild to moderate) injuries. Conversely, noxious stimulation may have little effect when applied after a severe injury that disrupts communication with rostral systems. Our last experiment evaluates this implication by testing the effect of nociceptive stimulation on the development of hemorrhage in rats that have undergone a light (6.25 mm drop), moderate (12 mm drop), or severe (25 mm drop) injury. A no injury (0 mm drop) control was also included. To verify that manipulating injury severity had the expected behavioral effect, and to assess whether shock treatment has an acute effect on motor performance, locomotor behavior was assessed before and after nociceptive stimulation was applied a day after injury.

As expected, rats that underwent a sham surgery (0 mm drop) exhibited unimpaired locomotor performance 24 h later [mean (±SE) BBB score = 20.92 ± 0.08]. Nociceptive stimulation had no effect on locomotor performance in uninjured animals (change in BBB score = 0.0). Likewise, histology revealed that neither the sham surgery or shock treatment induced signs of hemorrhage, with just one animal per condition exhibiting a small speck of hemorrhage-like staining (mean *%* = 0.00015 ± 0.00001).

**Figure 3:**
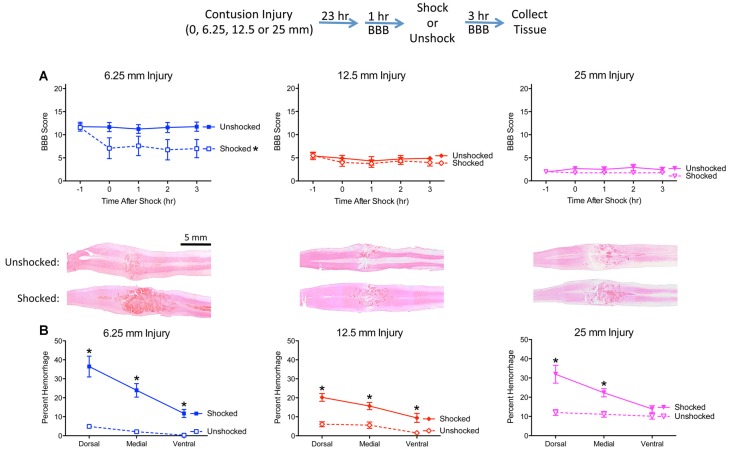
Noxious electrical stimulation has a more robust effect after a mild (6.25 mm) contusion injury. The experimental design is illustrated at the top of the figure. Rats received a sham surgery (0 mm), mild (6.25 mm), moderate (12.5 mm), or severe (25 mm) contusion injury. **(A)** Locomotor performance prior to nociceptive stimulation (−1 h) declined as injury severity increased. Noxious electrical stimulation (Shocked) induced an acute disruption in motor behavior, relative to the unstimulated (Unshock) controls, and this effect was most evident after a mild (6.25 mm) injury. **(B)** Nociceptive stimulation increased histological signs of hemorrhage and this effect was most evident within the dorsal region in animals that received a mild (6.25 mm) injury. Nociceptive stimulation did not affect locomotor scores, or induce signs of hemorrhage, in sham-operated animals. Representative sections are from the dorsal region (bar = 5 mm). Error bars indicate the SEM. *Indicates statistical significance (*p* < 0.05, *n* = 4).

Prior to nociceptive stimulation (−1 h), injured rats exhibited a disruption in locomotor performance and the magnitude of this effect increased with injury severity (Figure 3A). An ANOVA confirmed that injury severity had a statistically significant effect, *F*_(3,28)_ = 275.632, *p* < 0.0001. *Post hoc* comparisons showed that each severity condition differed from the other three (*p* < 0.05).

Nociceptive stimulation had no effect on locomotor scores in uninjured rats but led to an acute drop in motor behavior after injury. This effect was most evident after the least severe (6.25 mm) injury. An ANCOVA, using the baseline (−1 h) behavioral score as a covariate, confirmed that the effect of shock treatment, injury severity, and their interaction, were statistically significant, all *F*s > 6.076, *p* < 0.0034. *Post hoc* comparisons confirmed that shocked 6.25 mm injured rats differed from their unshocked (6.25 mm injured) controls (*p* < 0.05). Beyond this, group differences were determined by injury severity.

Histological analyses confirmed that the extent of tissue damage co-varied with injury severity (Figure 3B). Nociceptive stimulation increased the area of hemorrhage, and this effect was most evident in animals that received a 6.25 mm injury. A comparison of the level of hemorrhage observed along the dorsal-ventral axis revealed that shock treatment had a greater effect in the dorsal region (illustrated in Figure 3B). An ANOVA confirmed that the effect of shock treatment, injury severity, and their interaction, were statistically significant, all *F*s > 10.102, *p* < 0.0002 (all *η*^2^ > 0.153). The amount of hemorrhage varied across region, *F*_(2,48)_ = 48.649, *p* < 0.0001, and the magnitude of this effect depended upon shock treatment and injury severity, all *F*s > 3.924, *p* < 0.0029. The three-way interaction emerged because the effect of shock treatment on percent hemorrhage was most evident after a less severe (6.25 mm injury) in the dorsal and medial regions. To further analyze the nature of this three-way interaction, independent ANOVAs were performed on the dorsal, medial, and ventral sections. In all three cases, the effect of nociceptive stimulation depended upon injury severity, all *F*s > 4.74, *p* < 0.0098. In each case, the interaction emerged because the magnitude of the shock effect was inversely related to injury severity.

## Discussion

Prior work established that noxious electrical stimulation caudal to a spinal cord contusion injury impairs long-term recover (Grau et al., 2004). We have suggested that electrical stimulation has this effect because it engages pain fibers. Supporting this, it has been shown that the maladaptive consequences of shock treatment emerge at an intensity that engages C-fibers and sensitizes nociceptive systems (Baumbauer et al., 2008; Ferguson et al., 2008, 2012). More importantly, a treatment that selectively engages pain fibers (peripheral application of the irritant capsaicin) also impairs long-term recovery (Hook et al., 2008; Turtle et al., 2018). The adverse effect of noxious stimulation on tissue sparing has been related to the activation of cell signals that initiate cell death, the breakdown of the BSCB and hemorrhage at the site of injury (Garraway et al., 2014; Turtle et al., 2018, 2019). We previously assayed these effects within a 1 cm region of the spinal cord that encompassed the injury. Here we examined whether noxious stimulation has a broader effect that impacts tissue rostral or caudal to the site of injury. This issue was addressed by performing cellular assays, and histopathology, along a 3-cm segment of the spinal cord. As previously reported (Garraway et al., 2014; Turtle et al., 2018), noxious stimulation increased the content of alpha hemoglobin at the site of injury. It also elevated the expression of TNF, IL-1β, IL-18, and caspases 1 and 3. Statistical analyses show that the magnitude of the shock effect did not vary as a function of region for caspase 1, IL-1β, or IL-18, implying that stimulation had a relatively broad effect on these cellular signals. Western blotting for hemoglobin revealed some hemorrhage rostral to the site of injury. The latter observation was supported by histopathological analyses, which showed that the spread of hemorrhage was rostrally skewed. Hemorrhage may develop unevenly along the rostral-caudal axis because blood enters the damaged region largely from the rostral side.

It was originally hypothesized that uncontrollable noxious stimulation impairs long-term recovery after a contusion injury because this stimulation inhibits adaptive plasticity in spinally-transected animals (Grau et al., 1998, 2006; Crown et al., 2002). Research suggests that noxious stimulation has this effect because it sensitizes nociceptive circuits within the lumbosacral region (Ferguson et al., 2006, 2012; Grau, 2014; Huang et al., 2016). Given this, it was hypothesized that pain input caudal to a contusion injury would promote neural over-excitation within the lumbosacral tissue and that this effect could drive tissue loss at the site of injury (T11–12). From this perspective, one would anticipate a pattern of hemorrhage and proinflammatory cytokine expression that is skewed towards the caudal (lumbosacral) region. The present study found the opposite, providing a hint that other processes fuel the spread of secondary injury.

The parallel with work conducted in spinally transected rats also broke down when we tested the effect of transecting surviving fibers rostral to the contusion injury. Considerable evidence exists that descending fibers regulate the development of nociceptive sensitization in the lumbosacral spinal cord (Hains et al., 2003; Crown and Grau, 2005; Sandkühler, 2009; Huang and Grau, 2018), a process that helps to quell over-excitation. Supporting this, nociceptive stimulation does not induce a lasting modification within the lumbosacral spinal cord in uninjured (non-transected) rats (Gjerstad et al., 2001; Washburn et al., 2007). Based on these observations, we hypothesized that spared fibers have a protective effect that would lessen tissue loss in contused animals. This predicts that cutting communication with the brain, by means of a rostral (T2) transection, would remove a brake on pain input and amplify proinflammatory cytokine expression and hemorrhage. We found the opposite—disrupting communication with the brain blocked nociception-induced hemorrhage and proinflammatory cytokine expression. Recognizing the novelty of this finding, we sought converging evidence using histopathology to quantify the extent of hemorrhage. In addition, we addressed the possibility that the second surgery had a muting effect because it was performed 6 h before testing. To address this issue, the cord was transected at the time of the contusion injury. As expected, noxious stimulation increased the area of hemorrhage in non-transected animals and here too the effect was blocked by a rostral transection. Elsewhere we have shown that pharmacologically inhibiting communication with the brain, by slowly infusing the anesthetic lidocaine at T2, also blocks pain-induced hemorrhage (Davis et al., 2018). The latter suggests that these effects emerge because they disrupt communication with the brain and argue against interpretations based on a surgery-induced spinal shock (Bach-y-Rita and Illis, 1993; Dietz, 2010) or disruption in blood flow. Pain fibers are implicated in these effects because a rostral transection also blocks capsaicin-induced hemorrhage (Fauss et al., 2018). Taken together, the findings imply that the adverse effect of noxious stimulation on tissue sparing depends upon the engagement of a brain-dependent process. Transecting ascending fibers blocks the activation of these processes and prevents pain-induced hemorrhage and the spread of secondary injury within the spinal cord. This does not negate the idea that descending fibers can have a protective effect. That effect, though, is seemingly insufficient to counter a second brain-dependent process that fuels hemorrhage. To our knowledge, this is the first demonstration that rostral (brain systems) can drive tissue loss after SCI.

If the adverse effect of noxious stimulation applied caudal to injury depends upon ascending fibers and the activation of brain processes, the effect of pain input should interact with injury severity. The counter-intuitive prediction is that, the effect of noxious stimulation should be most evident after a mild to moderate injury; a severe injury should disrupt ascending fibers and lessen the activation of brain-dependent processes that fuel hemorrhage. The results of our last experiment were consistent with this prediction. In addition, the experiment showed that noxious stimulation induces an acute disruption in behavioral function after a mild contusion injury. The fact that locomotor performance declined immediately after stimulation suggests that pain input may trigger hemorrhage within minutes of application.

How could engaging brain processes affect tissue loss at the site of injury? One potential answer builds on the observation that pain input induces an acute rise in heart rate and blood pressure (Fauss et al., 2018). Other work has shown that a period of hypertension after injury is associated with poor prognosis (Nielson et al., 2015). Given this, we suggest that nociceptive input has a dual effect—it triggers processes at the site of injury that weaken the BSCB and, through brain-dependent processes, produces a rise in blood pressure that pushes blood into the surrounding tissue, expanding the area of injury. This hypothesis suggests caution is warranted during the acute period of clinical treatment, where the standard of care recommends maintaining systolic blood pressure above 85 mm Hg (Ryken et al., 2013; Inoue et al., 2014).

Other work has shown that noxious stimulation (e.g., colorectal distension) can induce a rise in heart rate and blood pressure in animals that have undergone a high thoracic transection (Weaver, 2002; Rabchevsky, 2006). These indices of autonomic dysreflexia typically emerge slowly, in the days to weeks after injury (Krassioukov et al., 2003). It could be argued that these same pathways mediate the acute effect of nociceptive stimulation on blood pressure in contused animals. We recently evaluated this possibility, showing that noxious stimulation caudal to a contusion injury induces an increase in systolic blood pressure and that this effect too is eliminated by a T2 transection (Fauss et al., 2018). These results suggest that the acute effect of noxious stimulation does not reflect a form of autonomic dysreflexia. It should be recognized, however, that the emergence of dysreflexia after injury may induce bouts of hypertension that could have a damaging effect (Weaver, 2002; Rabchevsky and Kitzman, 2011).

In summary, we detailed the rostral-caudal spread of pain induced hemorrhage, and cytokine expression, in contused animals. In the process, we discovered that the emergence of these effects depends on the integrity of spared fibers. The implication is that pain input engages a brain-dependent process that fuels hemorrhage at the site of SCI. The results are consistent with other work demonstrating that a number of cell signals (e.g., brain-derived neurotrophic factor, GABA, and dopamine) can have a bidirectional effect on nociceptive processing within the spinal cord (Sandkühler and Gruber-Schoffnegger, 2012; Huang et al., 2016, 2017; Puopolo, 2019). Further work is needed to uncover the fiber pathways and brain systems involved, the sensory/neurochemical triggers, and the systemic factors that contribute to the development of pain-induced hemorrhage.

## Data Availability

The datasets generated for this study are available on request to the corresponding author.

## Ethics Statement

The work has been reviewed and approved by our Institutional Animal Care and Use Committee. All of the authors have reviewed and approved the current text.

## Author Contributions

The study was conducted by JR and MH, who contributed equally to the work. JT and JG provided input on study design. RB and DJ assisted in the data collection. The data were analyzed by JR, MH, RB, DJ, and JG. The article was written by JR, MH, and JG, with input from JT, RB, and DJ.

## Conflict of Interest Statement

The authors declare that the research was conducted in the absence of any commercial or financial relationships that could be construed as a potential conflict of interest.
